# Editorial: Potential clinical applications of circulating microRNAs in neurosurgery

**DOI:** 10.3389/fsurg.2022.993898

**Published:** 2022-08-17

**Authors:** Ilgiz Gareev, Ozal Beylerli, Albert Sufianov, Daming Zhang

**Affiliations:** ^1^ Department of Neurosurgery, Federal Center of Neurosurgery, Tyumen, Russia; ^2^ Educational and Scientific Institute of Neurosurgery, Рeoples’ Friendship University of Russia (RUDN University), Moscow, Russian Federation; ^3^ Department of Neurosurgery, Sechenov First Moscow State Medical University (Sechenov University), Moscow, Russia; ^4^ Department of Neurosurgery, The First Affiliated Hospital of Harbin Medical University, Harbin, China

**Editorial on the Research Topic** Potential clinical applications of circulating microRNAs in neurosurgery By Gareev I, Beylerli O, Sufianov A, Zhang D. (2022) Front. Surg. 9: 993898. doi: 10.3389/fsurg.2022.993898

**Figure 1:**
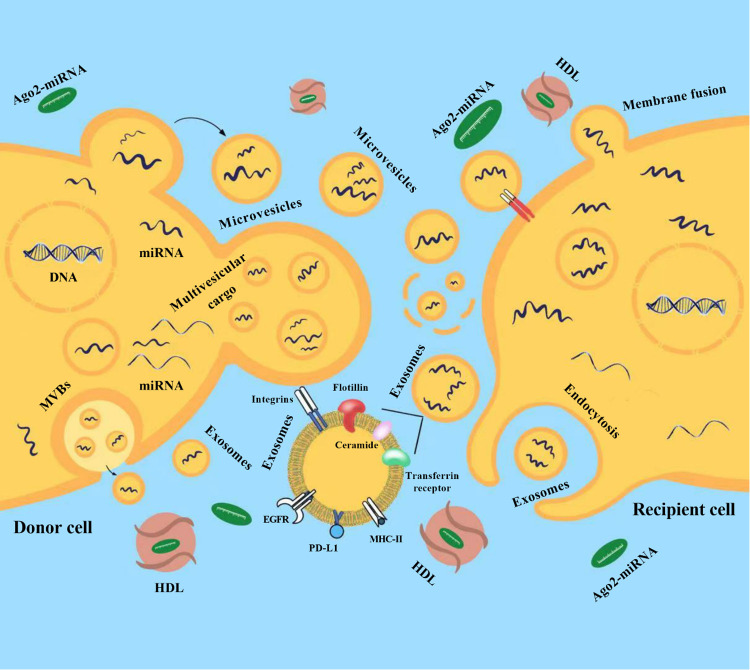
Release of microRNAs (miRNAs) into the extracellular environment. Released miRNAs are transported by extracellular vesicles (EVs) and transferred to recipient cells (from donor cells) where they regulate post-transcriptional gene expression. Membrane exosomes are released from cells as endosomes containing multivesicular bodies (MVBs) that fuse with the plasma membrane. Microvesicles (MVs) are released from the plasma membrane *via* outward budding. Multivesicular cargo (MVC) is released by membrane budding during apocrine secretion. EVs and their miRNAs cargo are transferred to recipient cells (from donor cells) after endocytosis or membrane fusion. MiRNAs can also be secreted outside of vesicles. Most circulating miRNAs are in a non-vesicular form, namely, they are associated with Ago2 proteins (Ago2-miRNA complex). In addition to the Ago2 protein, high-density lipoproteins (HDL) are reported to be involved in the mechanism of intercellular communication and are involved in the transport and delivery of miRNAs. EGFR, epidermal growth factor receptor; PD-L1, programmed death-ligand 1; MHC-II, major histocompatibility complex class II.

Neurosurgical pathology occupies a special place in surgical practice. As with other surgical specialties, this is a century-old specialization of the industry. In modern neurosurgery, for a qualitative approach to the treatment of patients with various diseases of the central nervous system (CNS), it is necessary to solve many diagnostic issues related to the peculiarities of this narrowly focused branch of medicine. One of the urgent problems in neurosurgery is the search for diagnostic and prognostic biomarkers for several neurosurgical pathologies, such as brain tumors or intracranial aneurysms with high risk rupture, the diagnostic methods of which currently require significant improvements. In modern neurosurgery, the search for predictive biomarkers, especially in brain tumors and cerebrovascular disease, is of paramount importance, since the introduction of these indicators into clinical practice will quickly determine the optimal treatment for each patient. Diagnosis plays a crucial role in making a prognosis and choosing the best therapy for brain tumors. Despite significant recent advances in the diagnosis of brain tumors using various modifications of imaging techniques followed by histopathological examination, tumor detection is still limited by its size and location, as well as by the heterogeneity of its tissue (1). In this regard, it is necessary to develop new diagnostic approaches that, together with the available methods, will improve the accuracy of diagnosis. A promising approach is fluid biopsy, which involves finding and measuring the levels of various circulating molecules in human body fluids such as blood or cerebrospinal fluid (CSF). In addition, given that computed tomography angiography (CTA), magnetic resonance angiography (MRA), and selective cerebral angiography (SCA) are either unavailable or do not provide clear evidence of possible rupture of intracranial aneurysms (IAs), accurate and reliable analysis of the molecular profile in biological fluids can help in the early diagnosis and prognosis of rupture, as well as likely mortality and morbidity or prognostic outcome of patients with subarachnoid hemorrhage (SAH) to stratify patients admitted to hospitals (1). MicroRNAs (miRNAs) are promising candidates for the role of such biomarkers. This is due to tissue specificity and a high rate of changes in miRNAs expression, combined with the possibility of these molecules leaving cells into the extracellular space/biological fluids in a form that is stable to degradation (Figure 1) (2).

Many studies have been published on the diagnostic significance of circulating miRNAs in primary and metastatic brain tumors, cerebrovascular disease (e.g., hemorrhage stroke and SAH), osteochondrosis, and traumatic brain injuries, etc. For instance, one prospective multi-center observational study identified a panel of 762 plasma miRNAs for each patient to establish these circulating miRNAs signatures specific to glioblastoma, and was capable of distinguishing them from malignant non-glial brain tumors (primary CNS lymphomas (PCNSL) and brain metastases (BM)) (NCT03630861, ClinicalTrials.gov). The EVTRNA study (NCT04230785, ClinicalTrials.gov) analyzed the differentiated expression pattern of circulating miRNAs by next-generation sequencing (NGS) in acute ischemic stroke (IS) patients before and/or after endovascular treatment. The candidate circulating miRNAs were verified as the biomarker and regulator for the progression and prognosis of acute IS with endovascular treatment.

Circulating miRNAs are attractive candidates for monitoring cerebrovascular disease (3). It is assumed that changes in expression of circulating microRNAs in biological fluids occur earlier than currently known biomarkers (e.g., markers of inflammation and repair, which include troponin, D-dimer, C-reactive protein, chemokines, and cytokines). Recently, a number of studies have been carried out, showing that circulating miRNAs may be more preferable as biomarkers since they can be detected early in the development of the disease, whereas protein structures like D-dimer and C-reactive protein are found in the blood only when a significant amount of damage has already occurred (4, 5). In addition, concentrations of D-dimer or C-reactive protein can also be increased in other pathologies, including thrombosis, infections, and myocardial infarction.

The stability and availability of circulating miRNAs in biological fluids make them new non-invasive biomarkers of particular interest in modern neurosurgery. The areas of application of circulating miRNAs will not only cover brain tumors or cerebrovascular disease but may also extend to many other neurosurgical pathologies. However, to minimize the variability of results, it is necessary to standardize sample processing procedures, detection methods, and, above all, normalization strategies. In addition, another breakthrough that we are now seeing is the study of miRNAs in extracellular vesicles such as exosomes, which are now available through various isolation methods, allowing them to be studied as biomarkers according to their cellular origin.
